# *Drosophila* SOCS Proteins

**DOI:** 10.1155/2011/894510

**Published:** 2011-12-13

**Authors:** Wojciech J. Stec, Martin P. Zeidler

**Affiliations:** MRC Centre for Developmental and Biomedical Genetics and Department of Biomedical Science, The University of Sheffield, Firth Court, Sheffield S10 2TN, UK

## Abstract

The importance of signal transduction cascades such as the EGFR and JAK/STAT pathways for development and homeostasis is highlighted by the high levels of molecular conservation maintained between organisms as evolutionary diverged as fruit flies and humans. This conservation is also mirrored in many of the regulatory mechanisms that control the extent and duration of signalling *in vivo*. One group of proteins that represent important physiological regulators of both EGFR and JAK/STAT signalling is the members of the SOCS family. Only 3 SOCS-like proteins are encoded by the *Drosophila* genome, and despite this low complexity, *Drosophila* SOCS proteins share many similarities to their human homologues. SOCS36E is both a target gene and negative regulator of JAK/STAT signalling while SOCS44A and SOCS36E represent positive and negative regulators of EGFR signalling. Here we review our current understanding of *Drosophila* SOCS proteins, their roles *in vivo,* and future approaches to elucidating their functions.

## 1. Introduction

**Figure 1:**
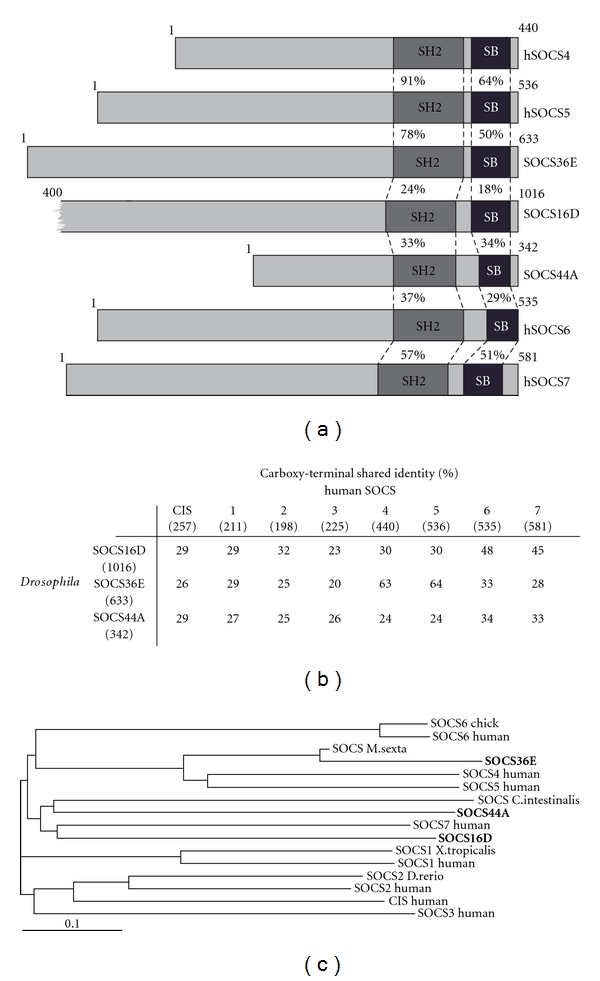
Structural conservation of SOCS family proteins. (a) Schematic representation of SOCS proteins. Percentage of conserved amino acids within the regions specified is shown as is protein sizes. Red indicates the SH2 domain and SOCS-box (SB) domain is shown in green. (b) Conservation of the carboxy-terminal regions (including the SH2 and SOCS-box domains) of human and *Drosophila* SOCS-family proteins is shown as percentage shared identity. Numbers in brackets indicate length of the full-length protein. (c) Phylogram representing common ancestry of full-length SOCS proteins from multiple species as indicated, *Drosophila* SOCS proteins are in bold. Identities and phylogram shown are generated by the ClustalW2 sequence alignment analysis tool [2].

Signalling pathways are required for correct development as well as maintenance of homeostasis in all multicellular organisms, while misregulation of these pathways is frequently associated with a range of diseases, including cancer and associated neoplasias. To avoid such events, multiple forms of regulation have emerged with essentially every level of most signalling cascades being targeted for regulation. To assure tight control of signalling output, families of specialised proteins have evolved that can function via mechanisms including sequestration of the pathway ligands, formation of inactive receptor complexes, inhibition of kinases, or regulation of transcriptional activity. The Suppressor of Cytokine Signalling (SOCS) family has been found to regulate JAK/STAT as well as receptor tyrosine kinase signalling such as the EGFR pathway. The mammalian family of SOCS proteins consists of eight members, SOCS1–7 and CIS (reviewed elsewhere in this issue and in [1]), and each contains a centrally located SH2 domain and a SOCS box situated in the C-terminus. SOCS4–7 are characterized by long dissimilar N-terminal regions lacking any distinct domains (Figure 1(a)). By contrast, SOCS1 and 3 have short N-terminal domains that contain a kinase inhibitory region located immediately upstream of the SH2 domain. All SOCS family members bind to phosphorylated tyrosine residues via their SH2 domains; this association allows SOCS proteins to bind to phosphorylated JAKs and receptors and may act as a direct steric inhibitor preventing Signal Transducer and Activator of Transcription (STAT) molecules from associating with the activated receptor/JAK complex [1]. In addition, interactions via the SH2 domain also provide a substrate recognition function for the SOCS-box associated Elongin-Cullin-SOCS (ECS) E3 ubiquitin ligase complex. In this scenario, the SOCS-box domain interacts with Elongins B and C, which in turn recruit Cullin 5 and Roc/Rbx1 to generate a competent Ubiquitin E3 ligase complex. Docking of this complex allows the transfer of ubiquitin moieties onto the substrate molecule, targeting it for degradation. 

While the biochemical interactions of human SOCS proteins are being progressively elucidated, the role of these proteins *in vivo* is less easily determined. One system in which SOCS proteins can be readily examined *in vivo* is the genetically tractable *Drosophila* model system. Recent developments from *Drosophila* regarding JAK/STAT, EGFR signaling, and SOCS regulation are discussed below.

## 2. JAK/STAT Pathway in *Drosophila*

The *Drosophila* JAK/STAT signalling pathway is stimulated by three Unpaired-like ligands, Upd [3], Upd2 [4], and Upd3 [5]. Ligand binding to a single transmembrane receptor, Domeless (Dome) [6], causes the activation of the associated JAK termed Hopscotch (Hop) [7]. Phosphorylation of both Hop and Dome subsequently leads to the binding of STAT92E [8, 9]. Following pathway stimulation, the STAT92E transcription factor becomes phosphorylated and translocates to the nucleus, where it induces transcription of pathway target genes [10–12] (reviewed in [13]). As such, conservation of pathway function between human and *Drosophila* systems is considerable despite lower redundancy compared to the mammalian system. *Drosophila* JAK/STAT signalling *in vivo *has been shown to be involved in multiple processes including embryonic patterning [8, 14], wing formation [15], migration of border cells during oogenesis [16, 17], maintenance of stem cells in stem cell niches [18–21], eye development [22], and immune responses [23, 24].

Given these diverse roles, it is not surprising that multiple regulators of JAK/STAT pathway signalling have also been conserved between vertebrates and *Drosophila*. One example is the tyrosine phosphatase PTP61F, identified by RNAi screening as a potent negative regulator of pathway signalling both *in* and *ex vivo* [25, 26]. *Drosophila* homologues of the vertebrate Protein Inhibitor of Activated STAT (PIAS) [27, 28] and the Signal Transduction Adaptor Molecule (STAM) [29] have also been characterised.

## 3. *Drosophila* SOCS Molecules

In addition to the JAK/STAT pathway regulators described above, three SOCS family members are encoded by the *Drosophila *genome and are termed SOCS16D, SOCS36E, and SOCS44A on the basis of their chromosomal location (Figure 1(a)) [30–32]. Sequence analysis reveals a conserved SOCS-typical domain structure, with SH2 and SOCS-box domains located in the carboxy-terminal (Figure 1(a)). As expected by analogy to vertebrate homologues, N-terminal regions do not show conservation. Based on the conserved carboxy-terminal region, SOCS36E is most homologous to hSOCS5, sharing 64% identity, and SOCS16D shows 48% and 45% identity to hSOCS6 and 7, respectively, while SOCS44A shares 34% and 33% identity with the same proteins, respectively (summarised in Figure 1(b)). The relationship of the three *Drosophila* SOCS-like proteins to mammalian SOCS proteins suggests common ancestry of SOCS16D and 44A, which is separate from SOCS36E. Strikingly, all *Drosophila *SOCS contain N-terminal regions at least 100 residues longer than hSOCS1-3, suggesting that the mammalian SOCS proteins with short N-termini may have arisen after divergence of mammals and insectas (Figure 1(c)).

While best studied in *Drosophila*, SOCS-like molecules have also been described in other invertebrate models including the moth, *Manduca sexta* [33], and the flour beetle, *Tribolium* [34].

## 4. *Drosophila* SOCS-Genes as Transcriptional Targets of JAK/STAT Pathway Signalling

**Figure 2:**
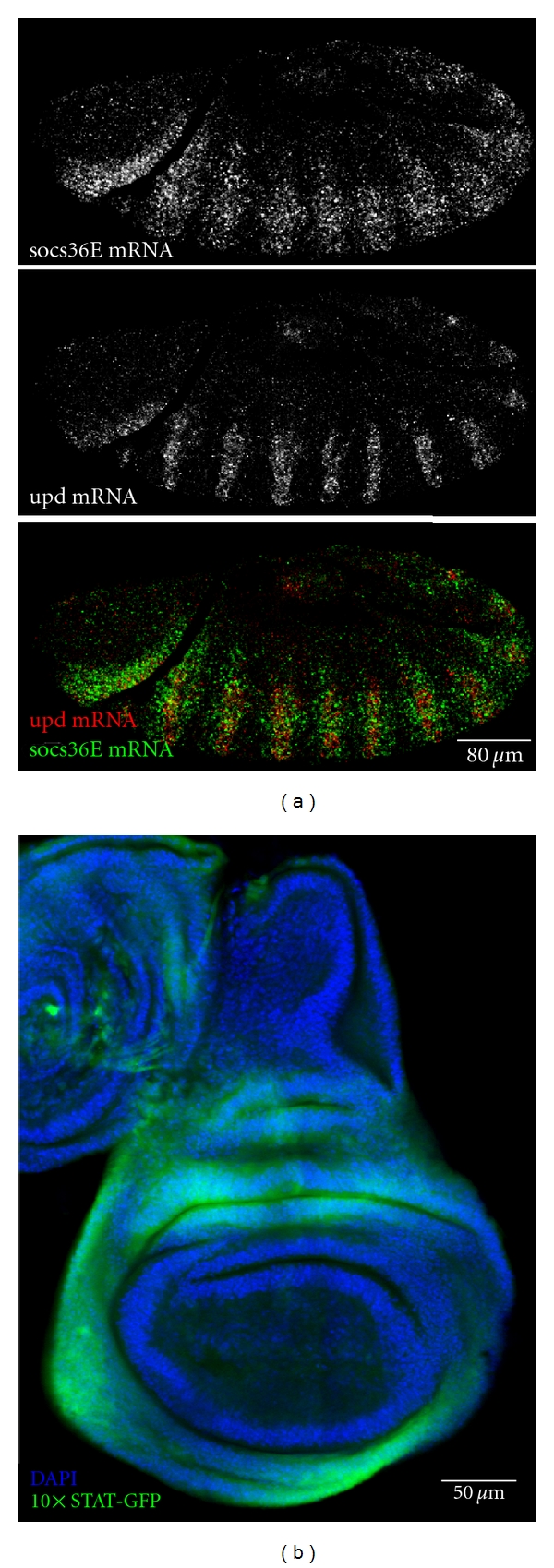
Expression of SOCS36E is a proxy for JAK/STAT pathway activity and can be used as a pathway reporter. (a) Double fluorescent *in situ* hybridization demonstrates the association between the expression domains of *upd *(top and red) and *socs36E *(middle and green) within a stage 13 embryo. (b) Late third instar wing imaginal disc expressing the *10xSTAT-GFP* reporter construct (green) in regions of high JAK/STAT activity that correspond to *upd *mRNA expression domains [37]. DNA (blue) outlines wing disc morphology.

The *socs36E* promoter region contains 19 putative STAT92E consensus binding sites and generates a corresponding mRNA expression pattern that closely mirrors Upd expression [31], a point highlighted by double fluorescent *in situ* hybridisation of *upd* and *socs36E* mRNA during embryogenesis (Figure 2(a)). Given this expression pattern, it appears that pathway downregulation elicited by SOCS36E acts as a classical negative feedback loop in a manner analogous to other vertebrate SOCS-family members [35]. Northern blot analysis has demonstrated strong expression of *socs36E* mRNA throughout embryogenesis, diminishing at later stages of development [30], a result in line with abundance of pathway ligands throughout early development. In flies lacking the Upd pathway ligands or the JAK kinase Hop, *socs36E* mRNA is largely absent [31, 32]. Conversely, mutant flies carrying the constitutively active kinase, Hop^Tuml^, or ectopically expressing Upd show increased levels of *socs36E* mRNA [31]. Cell culture studies have also demonstrated an increase in *socs36E* mRNA levels within 30 minutes of pathway stimulation and by 4 hours after stimulation, a 4.6-fold increase is detected compared to the initial expression suggesting that *socs36E* is a strong pathway target [12]. This fact has been utilised to generate a variety of *in vivo* and *ex vivo* reporters of JAK/STAT activity. These include the *10xSTAT-luciferase* reporter containing a pentamerised 441 bp region from the first intron of *socs36E* to generate a total of 10 potential STAT92E binding sites. This highly sensitive reporter has been used for an RNAi genomic screen [25], and a variant expressing GFP within transgenic *Drosophila* (termed *10xSTAT-GFP*) has also proven to be a powerful tool to report endogenous JAK/STAT pathway activity *in vivo* (Figure 2(b), [36]). 

By contrast, *socs44A* mRNA has not been identified as a transcriptional target of STAT92E [32] and neither *socs44A* nor *socs16D* is upregulated in transcript profiling experiments following pathway stimulation [12].

## 5. Regulation of the JAK/STAT Cascade

Although each of the three *Drosophila* SOCS-family proteins contains the SH2 and SOCS domains characteristic of SOCS regulators, only SOCS36E and SOCS44A have been found to regulate JAK/STAT pathway signalling, while limited studies on SOCS16D have not indicated any involvement with the JAK/STAT cascade [32]. In addition to cell-based studies that have used knockdown of *socs36E* as a control [5, 25, 26, 38], considerable analysis of the roles of SOCS proteins *in vivo *has also been undertaken.

The JAK/STAT pathway has a role in the development of *Drosophila* wings and their venation, which provides a convenient readout of the pathway activity [15]. Ectopic expression of SOCS36E in the developing wing results in an outstretched wing phenotype, analogous to that observed in regulatory *upd *mutants [30, 39]. Moreover, defects in venation of the wing were observed, consistent with mutants lacking *stat92E *and *hop*. Ectopic expression of SOCS44A also produces venation defects that do not completely phenocopy those achieved by misexpression of SOCS36E, suggesting that the two proteins may have different functions [32]. Genetic interaction experiments also suggest different roles for *socs36E* and *socs44A*. Increased dosage of SOCS44A in flies carrying combinations of weak loss-of-function Hop alleles results in increased lethality while ectopic expression of Hop leads to lethality that can be rescued by SOCS36E [30]. This indicates that SOCS36E is a strong negative regulator of the pathway while SOCS44A can suppress signalling to a weaker extent.

More detailed *in vivo *analysis of SOCS36E function comes from studies of the testicular stem cell niche. The testis stem cell niche is probably the best described niche to date and JAK/STAT pathway signalling has been shown to play a crucial role in stem cell maintenance within it [18, 19, 40]. Analysis of interactions between different components of the niche have also revealed a role for SOCS36E in maintaining the correct ratio of different stem cell populations within the niche [41]. In *socs36e* mutant testis a loss of germline stem cells (GSC) is observed in favour of somatic stem cells, termed Cist Progenitor Cells (CPC). Moreover, increased levels of STAT92E expression are observed in CPCs and cells of the hub upon removal of SOCS36E. Conversely, overexpression of SOCS36E in the testis leads to loss of CPCs but not GSCs, suggesting that SOCS36E negatively regulates maintenance and self-renewal of CPCs, allowing for GSC self-renewal [41].

Oogenesis is another well-studied process in which JAK/STAT pathway plays an important role. Besides maintaining the stem cell balance in the ovary niche in a manner analogous to the testis [42], pathway signalling has been shown to regulate migration of the border cells in the developing egg [16, 17, 43, 44]. Expression of Upd in the paired polar cells located at the anterior and posterior tips of the follicle results in recruitment of the adjacent follicular cells to form a cluster of presumptive border cells. Eight to ten cells will migrate along the midline of the egg chamber to meet the oocyte and form the micropyle, a sperm entry point [44–46]. Overexpression of SOCS36E in the border cells results in defects in recruitment and migration consistent with a reduction in JAK/STAT pathway activity [47]. SOCS44A has however not been found to be involved in oogenesis [32]. 

Flies carrying constitutively active Hop^Tuml^ develop haematopoietic abnormalities leading to formation of black melanised tumours [48]. Although the exact mechanism of tumour development has not been resolved, evidence for aberrant proliferation and differentiation of haemocyte precursors in the lymph gland (the *Drosophila *equivalent of a haematopoietic niche) exists [49, 50]. Overexpression of SOCS36E in the haemocyte precursors in the lymph gland is sufficient to produce a decrease in the number and size of tumours, while RNAi-mediated ablation of SOCS36E had the converse effect [12].

Despite the multiple strands of evidence demonstrating the role of SOCS36E as a negative regulator of the JAK/STAT pathway, it has to be noted that the null *socs36E *mutant allele is in fact homozygous viable [51, 52]. Considering the multiple requirements for JAK/STAT pathway signalling throughout development, this might seem counterintuitive. However, other pathway regulators of JAK/STAT signalling, including negative feedback loops, are known. These include the PTP61F phosphatase [25, 26], protein inhibitors of activated STAT (PIAS), and transcriptional repressors such as Ken and Barbie (reviewed in [13]). In addition, knockout of the mouse homologue of SOCS36E, SOCS5, is also homozygous viable, fertile, and does not display any phenotype [53]. As such, it appears likely that multiple forms of inhibition have emerged that are both evolutionary conserved and mutually redundant.

## 6. Regulation of EGFR Signalling

Wing venation requires JAK/STAT and EGFR/MAPK signalling pathways, that have been frequently found to cross-talk in mammals [32, 54–58]. The *Drosophila* EGFR pathway consists of four ligands (Gurken, Spitz, Argos, and Boss) that bind to three distinct receptors (DER, Torso, and Sevenless) and result in activation of the RAS-RAF-MAPK pathway (reviewed in [59]). The overall signalling pathway has been highly conserved across evolutionary time. In the mammalian system, SOCS4 and 5 negatively regulate EGFR signalling by targeting the receptor for degradation [60, 61]. As described above, ectopic expression of SOCS36E within the developing *Drosophila* wing produces venation defects in the adult wing which partially phenocopies loss of DER and suggests an inhibition of EGFR signalling [30]. The ability of SOCS36E to downregulate EGFR signalling is further supported by findings in the developing *Drosophila* eye. Specification of the eight photoreceptors (R1–R8) present within each ommatidial cluster requires intracellular signalling governed by EGFR signalling [62] with differentiation of the R7 receptor requiring an additional burst of signal in form of Sevenless (Sev) activation [62, 63]. EGFR receptor expression localizes to R1, R3, R4, R6, R7, and four ancillary cone cells, while SOCS36E is expressed in all cells with exception of R2, R5, and R7 [52]. In a *socs36E* mutant additional R7 receptors are recruited, while overexpression of SOCS36E is sufficient to prevent R7 cell differentiation. This demonstrates a requirement for SOCS36E in regulation of fate determination in the developing eye, a cell fate decision that does not involve JAK/STAT signalling [64]. Furthermore, misexpression of downstream components of the EGFR pathway together with SOCS36E also resulted in recruitment of additional R7 cells, indicating direct and specific interaction between SOCS36E and Sev. It has however been suggested that SOCS36E is only a weak repressor of Sev as high levels of Sevenless signalling is able to suppress the phenotypes caused by SOCS36E expression [52]. Results obtained in the wing and eye imaginal discs suggest that SOCS36E is also able to weakly inhibit EGFR pathway in these other tissues demonstrating a conserved function across species.

In addition to the role of SOCS36E, SOCS44A has also been shown to play a role in the regulation of EGFR signalling. Misexpression of SOCS44A in the developing wing produces venation defects similar to JAK/STAT loss of function as well as EGFR gain of function. Indeed, phenotypes characteristic for heterozygous mutations in *ras85D *and *EGFR* were rescued upon SOCS44A overexpression and enhanced by loss of *argos*, a negative regulator of the EGFR pathway. On this basis, as well as interactions between misexpressed *argos* and a genetic deficiency removing *socs44A*, it has been concluded that SOCS44A upregulates EGFR signalling in the wing [32]. However, studies in the developing eye failed to identify SOCS44A as a regulator of the EGFR pathway [52]. Considering that the presence of different EGF-like receptors is present in both tissues, these results suggest that SOCS44A may show specificity to a particular receptor. However, studies in mammalian systems suggest a different function for the SOCS44A homologue, SOCS6, which downregulates the EGFR receptor c-KIT by targeting it for degradation [65]. Ultimately, the precise interactions of *Drosophila* SOCS proteins in regulating both EGFR and JAK/STAT pathway signalling will require further analysis at both the genetic and biochemical levels.

## 7. Structural Analysis of SOCS36E

Multiple biochemical and structure-function analyses of mammalian SOCS proteins have revealed a range of different mechanisms by which they exert their pathway regulatory functions. To date, no such studies have been performed on *Drosophila *SOCS proteins; however, genetic analysis has highlighted the importance of the SH2 domain for correct function of SOCS36E. Ectopic expression of a protein carrying a point mutation within the SH2 domain previously shown to abolish interactions with phosphorylated tyrosine did not produce any phenotypes [30, 47, 52]. These results were not surprising considering the homology of SOCS36E to SOCS5 which has also been shown to require both the SH2 and SOCS-box domains for its function [61]. However, ectopic expression of a SOCS-box truncation of SOCS36E is sufficient to generate a wing vein phenotype that resembles the milder phenotypes generated by the wild type protein [30]. Misexpression of SOCS-box truncation is also sufficient to cause mild border cell migration defects and a decrease in ommatidial R7 cell frequency [47, 52]. Despite the lack of identifiable domains in the N-terminal region of both proteins, it seems likely that SOCS36E is able to regulate JAK/STAT signalling in a SOCS-box independent manner, possibly via competitive binding to the phosphorylated tyrosine. The structure-function relationship of SOCS44A remains to be addressed.

## 8. Conclusions

Signalling pathways require tight regulation to prevent outcomes harmful for development and maintenance of the organism. Acting in a context-specific manner negative regulators, like SOCS family of proteins, often act to fine-tune the signal adding to the robustness of the signal transduction pathways. Moreover, from systems biology perspective negative regulators can be viewed as integral components of the developmental machinery, allowing for precise regulation of cell fate specification, survival and death, among many other outcomes. Furthermore, multiple levels of negative regulation also introduce redundancies into the system, and as a result only mild phenotypes are observed following the loss of any one regulatory component.

**Figure 3:**
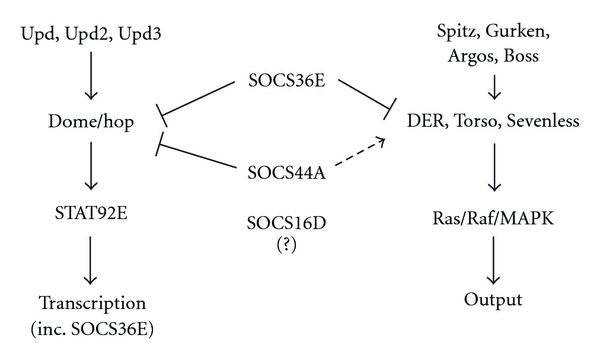
Schematic representation of the interaction of SOCS proteins with the JAK/STAT and EGFR pathways. Positive regulation indicated by arrows and negative regulation represented by blunt-ended arrows. Dashed line arrow indicates context-specific positive regulation.

Of the three SOCS proteins encoded by the *Drosophila *genome, SOCS36E and SOCS44A have been found to interact in different directions with both the JAK/STAT and EGFR signalling pathways (summarised in Figure 3). Homologous to mammalian SOCS5, SOCS36E has received much more attention than the two remaining fruit fly family members. Genetic as well as cellular studies have identified roles in development, spermatogenesis, oogenesis, and tumour development, establishing SOCS36E as a potent, yet redundant negative regulator of JAK/STAT pathway. Weak inhibition of EGFR signalling further indicates strong conservation of function across species. The ability of SOCS36E to negatively regulate JAK/STAT pathway activities following SOCS-box domain truncations indicates a possible additional mechanism of inhibition. It will be interesting to address the potential role of SOCS36E in the regulation of tumour formation in Hop^Tuml^ flies, a system previously shown to be a good model of *Drosophila* leukaemia and tumourigenesis studies.

SOCS44A has not yet been studied in detail. However our current understanding indicates its ability to weakly inhibit JAK/STAT pathway and positively regulate EGFR pathway, in a context-specific manner. This is in contrast to the function of SOCS6, the closest mammalian homologue of SOCS44A. Further studies on SOCS44A as well as SOCS16D will undoubtedly identify novel roles for the wider *Drosophila* SOCS family. Ultimately, the mutual *in vivo* interprotein relationships of the fly SOCS proteins might facilitate our understanding of the higher complexity mammalian SOCS protein interactions. 

## References

[1] Croker BA, Kiu H, Nicholson SE (2008). SOCS regulation of the JAK/STAT signalling pathway. *Seminars in Cell and Developmental Biology*.

[2] Chenna R, Sugawara H, Koike T (2003). Multiple sequence alignment with the Clustal series of programs. *Nucleic Acids Research*.

[3] Harrison DA, McCoon PE, Binari R, Gilman M, Perrimon N (1998). Drosophila unpaired encodes a secreted protein that activates the JAK signaling pathway. *Genes and Development*.

[4] Hombría JCG, Brown S, Häder S, Zeidler MP (2005). Characterisation of Upd2, a Drosophila JAK/STAT pathway ligand. *Developmental Biology*.

[5] Wright VM, Vogt KL, Smythe E, Zeidler MP (2011). Differential activities of the Drosophila JAK/STAT pathway ligands Upd, Upd2 and Upd3. *Cellular Signalling*.

[6] Brown S, Hu N, Hombría JCG (2001). Identification of the first invertebrate interleukin JAK/STAT receptor, the Drosophila gene domeless. *Current Biology*.

[7] Binari R, Perrimon N (1994). Stripe-specific regulation of pair-rule genes by hopscotch, a putative Jak family tyrosine kinase in Drosophila. *Genes and Development*.

[8] Hou XS, Melnick MB, Perrimon N (1996). Marelle acts downstream of the Drosophila HOP/JAK kinase and encodes a protein similar to the mammalian STATs. *Cell*.

[9] Yan R, Small S, Desplan C, Dearolf CR, Darnell JE (1996). Identification of a Stat gene that functions in Drosophila development. *Cell*.

[10] Karsten P, Plischke I, Perrimon N, Zeidler MP (2006). Mutational analysis reveals separable DNA binding and trans-activation of Drosophila STAT92E. *Cellular Signalling*.

[11] Flaherty MS, Zavadil J, Ekas LA, Bach EA (2009). Genome-wide expression profiling in the Drosophila eye reveals unexpected repression of Notch signaling by the JAK/STAT pathway. *Developmental Dynamics*.

[12] Bina S, Wright VM, Fisher KH, Milo M, Zeidler MP (2010). Transcriptional targets of Drosophila JAK/STAT pathway signalling as effectors of haematopoietic tumour formation. *EMBO Reports*.

[13] Arbouzova NI, Zeidler MP (2006). JAK/STAT signalling in Drosophila: insights into conserved regulatory and cellular functions. *Development*.

[14] Small S, Blair A, Levine M (1996). Regulation of two pair-rule stripes by a single enhancer in the Drosophila embryo. *Developmental Biology*.

[15] Yan R, Luo H, Darnell JE, Dearolf CR (1996). A JAK-STAT pathway regulates wing vein formation in Drosophila. *Proceedings of the National Academy of Sciences of the United States of America*.

[16] Ghiglione C, Devergne O, Georgenthum E (2002). The Drosophila cytokine receptor Domeless controls border cell migration and epithelial polarization during oogenesis. *Development*.

[17] Silver DL, Montell DJ (2001). Paracrine signaling through the JAK/STAT pathway activates invasive behavior of ovarian epithelial cells in Drosophila. *Cell*.

[18] Kiger AA, Jones DL, Schulz C, Rogers MB, Fuller MT (2001). Stem cell self-renewal specified by JAK-STAT activation in response to a support cell cue. *Science*.

[19] Tulina N, Matunis E (2001). Control of stem cell self-renewal in Drosophila spermatogenesis by JAK-STAT signaling. *Science*.

[20] Liu W, Singh SR, Hou SX (2010). JAK-STAT is restrained by Notch to control cell proliferation of the drosophila intestinal stem cells. *Journal of Cellular Biochemistry*.

[21] Wang W, Li Y, Zhou L, Yue H, Luo H (2011). Role of JAK/STAT signaling in neuroepithelial stem cell maintenance and proliferation in the Drosophila optic lobe. *Biochemical and Biophysical Research Communications*.

[22] Bach EA, Vincent S, Zeidler MP, Perrimon N (2003). A sensitized genetic screen to identify novel regulators and components of the Drosophila janus kinase/signal transducer and activator of transcription pathway. *Genetics*.

[23] Agaisse H, Perrimon N (2004). The roles of JAK/STAT signaling in Drosophila immune responses. *Immunological Reviews*.

[24] Kwon EJ, Park HS, Kim YS (2000). Transcriptional regulation of the Drosophila raf proto-oncogene by drosophila STAT during development and in immune response. *Journal of Biological Chemistry*.

[25] Baeg GH, Zhou R, Perrimon N (2005). Genome-wide RNAi analysis of JAK/STAT signaling components in Drosophila. *Genes and Development*.

[26] Müller P, Kuttenkeuler D, Gesellchen V, Zeidler MP, Boutros M (2005). Identification of JAK/STAT signalling components by genome-wide RNA interference. *Nature*.

[27] Betz A, Lampen N, Martinek S, Young MW, Darnell JE (2001). A Drosophila PIAS homologue negatively regulates stat92E. *Proceedings of the National Academy of Sciences of the United States of America*.

[28] Hari KL, Cook KR, Karpen GH (2001). The Drosophila Su(var)2-10 locus regulates chromosome structure and function and encodes a member of the PIAS protein family. *Genes and Development*.

[29] Mesilaty-Gross S, Reich A, Motro B, Wides R (1999). The Drosophila STAM gene homolog is in a tight gene cluster, and its expression correlates to that of the adjacent gene ial. *Gene*.

[30] Callus BA, Mathey-Prevot B (2002). SOCS36E, a novel Drosophila SOCS protein, suppresses JAK/STAT and EGF-R signalling in the imaginal wing disc. *Oncogene*.

[31] Karsten P, Häder S, Zeidler MP (2002). Cloning and expression of Drosophila SOCS36E and its potential regulation by the JAK/STAT pathway. *Mechanisms of Development*.

[32] Rawlings JS, Rennebeck G, Harrison SMW, Xi R, Harrison DA (2004). Two Drosophila suppressors of cytokine signaling (SOCS) differentially regulate JAK and EGFR pathway activities. *BMC Cell Biology*.

[33] Elliott GC, Zeidler MP (2008). MsSOCS expression indicates a potential role for JAK/STAT signalling in the early stages of Manduca sexta spermatogenesis. *Insect Molecular Biology*.

[34] Bäumer D, Trauner J, Hollfelder D, Cerny A, Schoppmeier M (2011). JAK-STAT signalling is required throughout telotrophic oogenesis and short-germ embryogenesis of the beetle Tribolium. *Developmental Biology*.

[35] Starr R, Willson TA, Viney EM (1997). A family of cytokine-inducible inhibitors of signalling. *Nature*.

[36] Bach EA, Ekas LA, Ayala-Camargo A (2007). GFP reporters detect the activation of the Drosophila JAK/STAT pathway in vivo. *Gene Expression Patterns*.

[37] Mukherjee T, Hombría JC, Zeidler MP (2005). Opposing roles for Drosophila JAK/STAT signalling during cellular proliferation. *Oncogene*.

[38] Vidal OM, Stec W, Bausek N, Smythe E, Zeidler MP (2010). Negative regulation of Drosophila JAK-STAT signalling by endocytic trafficking. *Journal of Cell Science*.

[39] Müller HJ (1930). Types of visible variations induced by X-rays in Drosophila. *Journal of Genetics*.

[40] Leatherman JL, Dinardo S (2008). Zfh-1 controls somatic stem cell self-renewal in the Drosophila testis and nonautonomously influences germline stem cell self-renewal. *Cell Stem Sell*.

[41] Singh SR, Zheng Z, Wang H, Oh SW, Chen X, Hou SX (2010). Competitiveness for the niche and mutual dependence of the germline and somatic stem cells in the Drosophila testis are regulated by the JAK/STAT signaling. *Journal of Cellular Physiology*.

[42] Decotto E, Spradling AC (2005). The Drosophila ovarian and testis stem cell niches: similar somatic stem cells and signals. *Developmental Cell*.

[43] Beccari S, Teixeira L, Rorth P (2002). The JAK/STAT pathway is required for border cell migration during Drosophila oogenesis. *Mechanisms of Development*.

[44] Xi R, McGregor JR, Harrison DA (2003). A gradient of JAK pathway activity patterns the anterior-posterior axis of the follicular epithelium. *Developmental Cell*.

[45] Grammont M, Irvine KD (2002). Organizer activity of the polar cells during Drosophila oogenesis. *Development*.

[46] Montell DJ (2003). Border-cell migration: the race is on. *Nature Reviews Molecular Cell Biology*.

[47] Silver DL, Geisbrecht ER, Montell DJ (2005). Requirement for JAK/STAT signaling throughout border cell migration in Drosophila. *Development*.

[48] Luo H, Hanratty WP, Dearolf CR (1995). An amino acid substitution in the Drosophila hop(Tum-l) Jak kinase causes leukemia-like hematopoietic defects. *EMBO Journal*.

[49] Krzemień J, Dubois L, Makki R, Meister M, Vincent A, Crozatier M (2007). Control of blood cell homeostasis in Drosophila larvae by the posterior signalling centre. *Nature*.

[50] Makki R, Meister M, Pennetier D (2010). A short receptor downregulates JAK/STAT signalling to control the Drosophila cellular immune response. *PLoS Biology*.

[51] Bellen HJ, Levis RW, Liao G (2004). The BDGP gene disruption project: single transposon insertions associated with 40% of Drosophila genes. *Genetics*.

[52] Almudi I, Stocker H, Hafen E, Corominas M, Serras F (2009). SOCS36E specifically interferes with sevenless signaling during Drosophila eye development. *Developmental Biology*.

[53] Brender C, Columbus R, Metcalf D (2004). SOCS5 is expressed in primary B and T lymphoid cells but is dispensable for lymphocyte production and function. *Molecular and Cellular Biology*.

[54] Heinrich PC, Behrmann I, Haan S, Hermanns HM, Müller-Newen G, Schaper F (2003). Principles of interleukin (IL)-6-type cytokine signalling and its regulation. *Biochemical Journal*.

[55] Shuai K, Liu B (2003). Regulation of JAK-STAT signalling in the immune system. *Nature Reviews Immunology*.

[56] Rane SG, Reddy EP (2000). Janus kinases: components of multiple signaling pathways. *Oncogene*.

[57] de Celis JF, Diaz-Benjumea FJ (2003). Developmental basis for vein pattern variations in insect wings. *International Journal of Developmental Biology*.

[58] Shilo BZ (2003). Signaling by the Drosophila epidermal growth factor receptor pathway during development. *Experimental Cell Research*.

[59] Simon MA (2000). Receptor tyrosine kinases: specific outcomes from general signals. *Cell*.

[60] Bullock AN, Rodriguez MC, Debreczeni JE, Songyang Z, Knapp S (2007). Structure of the SOCS4-elonginB/C complex reveals a distinct SOCS box interface and the molecular basis for SOCS-dependent EGFR degradation. *Structure*.

[61] Kario E, Marmor MD, Adamsky K (2005). Suppressors of cytokine signaling 4 and 5 regulate epidermal growth factor receptor signaling. *Journal of Biological Chemistry*.

[62] Freeman M (1996). Reiterative use of the EGF receptor triggers differentiation of all cell types in the Drosophila eye. *Cell*.

[63] Simon MA, Bowtell DDL, Dodson GS, Laverty TR, Rubin GM (1991). Ras1 and a putative guanine nucleotide exchange factor perform crucial steps in signaling by the sevenless protein tyrosine kinase. *Cell*.

[64] Zeidler MP, Perrimon N, Strutt DI (1999). Polarity determination in the Drosophila eye: a novel role for unpaired and JAK/STAT signaling. *Genes and Development*.

[65] Zadjali F, Pike ACW, Vesterlund M (2011). Structural basis for c-KIT inhibition by the suppressor of cytokine signaling 6 (SOCS6) ubiquitin ligase. *Journal of Biological Chemistry*.

